# Secular trends of vitamin D and calcium intake and their circulating levels in US adults from 2007 to 2018

**DOI:** 10.3389/fnut.2025.1538019

**Published:** 2025-03-19

**Authors:** Yongliang Du, Chao Ma, Zhaoyue Shang, Xiaohua Zhang, Yanlin Duan, Tong Liu, Yang Yu, Shuman Yang

**Affiliations:** ^1^Department of Endocrinology, The First Affiliated Hospital of Jinzhou Medical University, Jinzhou, China; ^2^Department of Epidemiology and Biostatistics, Jilin University, Jilin, China

**Keywords:** secular trends, vitamin D, calcium, serum total calcium, serum 25(OH)D

## Abstract

**Background:**

Few studies have examined the secular trends of total calcium and vitamin D intake and their circulating levels together among adults in the United States (US). Understanding the trends of these nutrients may be useful for refining existing nutrition policy and guidelines.

**Objective:**

The aim of this study was to report trends in total calcium and vitamin D intake and their circulating levels in the US population aged 18 years or older in 2007–2018.

**Methods:**

This cross-sectional study identified adults aged 18 years or older in the US National Health and Nutrition Examination Survey (NHANES) from 2007 to 2018. Calcium and vitamin D intake including their supplements were the average of two 24-h recalls. Serum calcium and serum 25-hydroxyvitamin D [25(OH)D] were measured using established methods. Weighted regression was used to test trends in calcium and vitamin D intake, and serum total calcium and 25(OH)D levels.

**Results:**

This research included 16,751 participants, including 9,046 males and 7,705 females. Serum total calcium significantly decreased with survey years from 2007 to 2018 (9.42 to 9.31 mg/dL) (*P* trend <0.001). Calcium intake declined from 2009 to 2018 (1,070 to 1,010 mg/day; *P* trend <0.001). In contrast, vitamin D intake and serum 25(OH)D increased with survey years (5.8 to 11.0 mcg/day and 65.6 to 68.5 nmol/L, respectively; all *P* trend <0.001). The trends in calcium intake vs. serum total calcium (*P* trend interaction =0.267), and vitamin D intake vs. serum 25(OH)D with survey years were comparable (*P* trend interaction =0.190). Inadequate vitamin D intake decreased with survey years (86.0 to 80.2%; *P* trend = 0.002). Moderate vitamin D deficiency (22.3 to 21.5%; *P* trend = 0.043), but not severe vitamin D deficiency (3.3 to 2.9%; *P* trend = 0.119), also declined with calendar years.

**Conclusion:**

From 2007 to 2018, US adults showed a decrease in serum total calcium, and an increase in serum 25(OH)D levels. Both trends were partly due to declined calcium and increased vitamin D intake.

## 1 Introduction

Calcium and vitamin D are important nutrients for maintaining human health. Calcium plays a vital role in maintaining the health of bones and teeth, mediating vascular contraction and vasodilatation, muscle function, nerve transmission, intracellular signaling, and hormonal secretion (1, 2). Vitamin D plays a crucial role in cell cycle regulation, muscle function, insulin signaling, and immune modulation, in addition to bone metabolism (3–5). The increased calcium intake from dietary sources slightly increased bone mineral density (BMD) at most sites over 1 to 2 years (an increase of 0.6–1.8%) (6). The reduced risk of ovarian cancer was related to a 100 mg/d increase in dietary calcium intake and a 100 IU/day increase in vitamin D intake (7). Calcium and vitamin D play an important role in the development of many diseases such as obesity, cancer, diabetes, and osteoporosis (8–10).

Few studies have examined the secular trends of calcium and vitamin D simultaneously. Existing studies focused on either calcium or vitamin D alone (11–13). For example, Rooney et al. only investigated the calcium supplementation from 1999 to 2014 (14). The trends of vitamin D supplements for years 1999–2012 had been examined in a United States (US) (15).

Bioactive vitamin D promotes intestinal calcium absorption, and renal tubular reabsorption of calcium and regulates bone homeostasis. Considering the combined effects of vitamin D and calcium, this research investigated the secular trends of total vitamin D and calcium intake, and their circulating levels among US adults. Through the regulation of parathyroid hormone, 25(OH)D and ionized calcium itself, the circulating calcium normally maintains within a tight physiological range (16). However, previous studies have reported that higher serum calcium levels, even within the normal range, are associated with abnormal vascular function, such as increased carotid intima–media thickness, arterial stiffness and the presence of calcified plaque (17).

Understanding the secular trends of vitamin D and calcium is useful for refining existing nutrition policy and guideline, and eventually improving the population health. Until now, there were no major adjustments for vitamin D and calcium intake following the creation of 2011 recommended dietary allowance (RDA) advices (18). However, the consensus on vitamin D intake and optimal serum 25(OH)D concentrations is still lacking due to the clinical trials and meta-analyses failed to find a beneficial effects of vitamin D on cardiovascular and cancer outcomes (19). There is still a concern about calcium intake and cardiovascular health (20). Thus, the recommended dietary allowance for vitamin D and calcium may need to be adjusted.

## 2 Methods

### 2.1 Study design and participants

The data for this study were extracted from the NHANES, conducted by the National Center for Health Statistics (NCHS) of the Centers for Disease Control and Prevention (CDC). All participants provided written informed consent. The NHANES data included demographics data, dietary data, examination data, laboratory data, questionnaire data, and limited access data; the data were collected through interviews and direct standardized physical examinations in equipped mobile examination centers using a complex, stratified, multistage probability sample.

**Figure 1:**
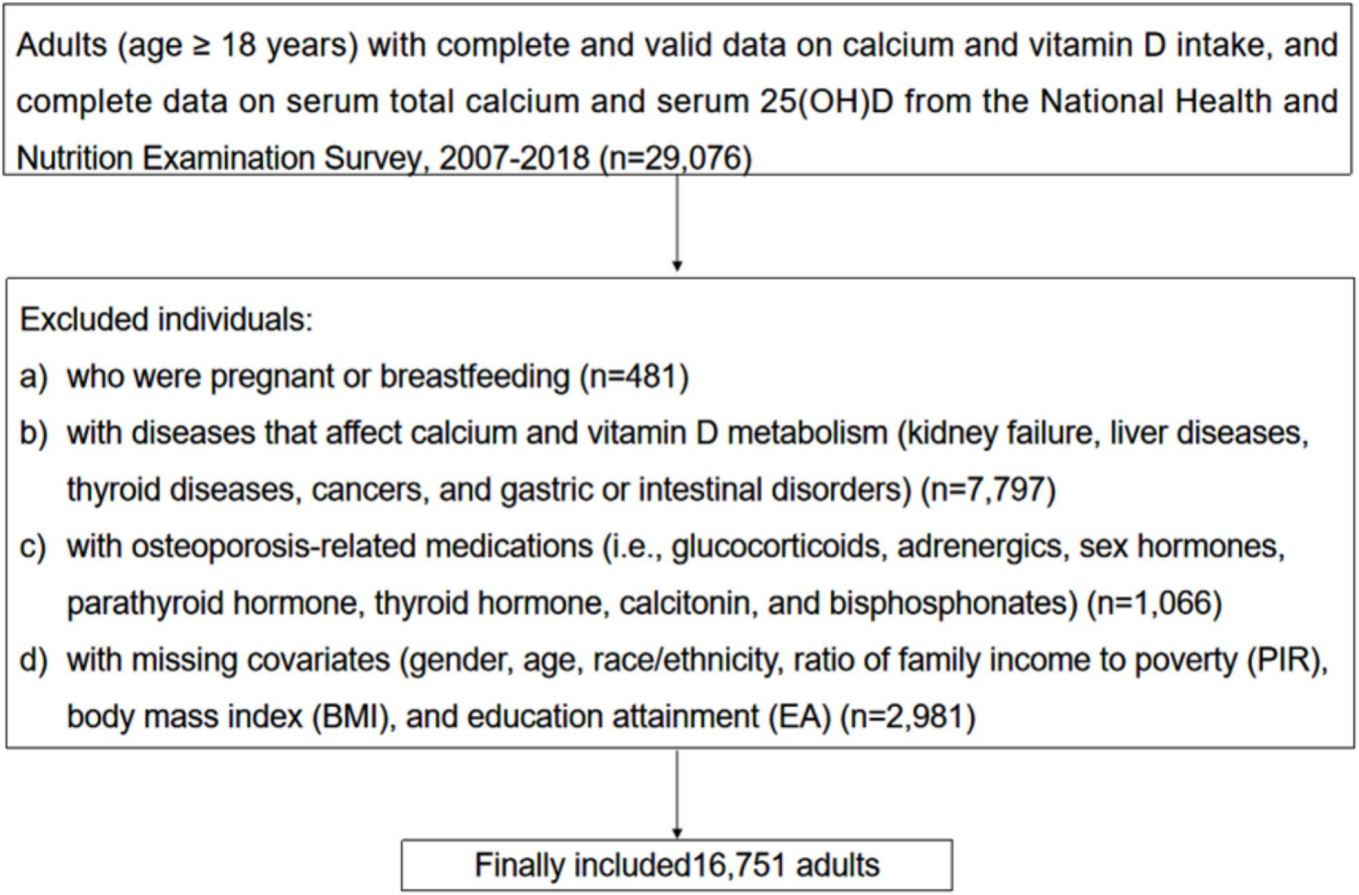
Flowchart of the individuals’ inclusion and exclusion criteria.

Based on the NHANES data from 2007 to 2018, we identified participants aged 18 years or older with complete and valid data on calcium intake, vitamin D intake, serum total calcium, and serum 25(OH)D (Figure 1). Valid calcium and vitamin D intake data were defined as reliable 24-h recalls for dietary data. We excluded participants who were pregnant or breastfeeding, and participants with diseases that affect calcium and vitamin D metabolism (e.g., kidney failure, liver problems, thyroid problems, cancers, gastric or intestinal disorders), with prescribed osteoporosis-related medications (i.e., glucocorticoids, adrenergics, sex hormones, parathyroid hormone, thyroid hormone, calcitonin, bisphosphonates), and with missing covariates.

### 2.2 Calcium and vitamin D intake

Data on calcium and vitamin D intake were assessed through two 24-h recalls. The first interview was conducted in the Mobile Examination Center (MEC); the second interview was conducted by telephone interview between 3 and 10 days after the first interview. Both interviews offered data on calcium and vitamin D intake. Conducting two interviews may offer more reliable information about habitual patterns of nutrient intake. A standard set of measurement guidelines, including 2-dimensional (2D) and 3-dimensional (3D), were used to help the respondent report the volume and dimensions of the food consumed. The 24-h recall followed the 5-step “automated multiple-pass method (AMPM),” which could enhance the completeness and accuracy of the food recall and reduce respondent burden. We calculated the mean of total calcium and vitamin D intake (dietary intake and supplements) using data from two 24-h recall interviews. The nutrient residual model was used to adjust total energy intake (21). The Estimated Average Requirement (EAR) for calcium and vitamin D, established by the Institute of Medicine in 2010, is an intake value used for assessing population-level nutritional adequacy (22). The EAR for calcium is 1,100 mg/day for people aged 18 years and under, 800 mg/day for men aged 19–70 years and women aged 19–50 years, and 1,000 mg/day for men >70 years and women >50 years. The EAR for vitamin D is 10 μg (400 IU) for people aged 1 year or above 20 years. We used EAR to determine the prevalence of participants with adequate calcium and vitamin D intake. The EAR-based grouping only considered the actual absolute nutrient intake per day, and did not adjust for total energy intake.

### 2.3 Measurement of circulating calcium and vitamin D

Blood samples were collected with the vacuum blood collection tube. The blood samples collected during the examination were centrifuged, and serum samples were divided into aliquots and shipped on dry ice to a variety of contact laboratories at which they were frozen until analysis.

Standardized liquid chromatography–tandem mass spectrometry (LC–MS/MS) method was used for the quantitative detection of serum 25(OH)D including 25-hydroxyvitamin D3 [25(OH)D3], epi-25-hydroxyvitamin D3 [epi-25(OH)D3], and 25-hydroxyvitamin D2 [25(OH)D2] from 2007 onwards (23). The coefficients of variation for serum 25(OH)D were ≤ 6.8%. The Beckman Synchron LX20 in 2007–2008 and the Beckman Coulter UniCel^®^ DxC800 system in 2009–2016 measured serum calcium concentration using indirect (or diluted) ISE (ion selective electrode) methodology. During 2017–2018, the Roche Cobas 6,000 Chemistry Analyzer measured serum calcium by spectrophotometric method. In this method, calcium reacted with 5-nitro-5’methyl-BAPTA under alkaline conditions to form a complex. This complex then reacted with EDTA (Ethylene Diamine Tetraacetic Acid) to form-colored products whose intensity was directly proportional to the concentration of calcium in the specimen. It is measured photometrically at 340 nm (24). The coefficients of variation for serum calcium measurements for both methods were ≤ 2.8%. Generally, the proportion of individuals with intakes below the EAR can reasonably estimate the expected incidence of inadequate intake in the population. According to previous guidelines and reports, 25(OH)D < 25.0 nmol/L was defined as severe vitamin D deficiency, 25.0–49.9 nmol/L as moderate deficiency, 50.0–74.9 nmol/L as insufficiency, ≥75.0 nmol/L as sufficiency (25–27). The referent serum total calcium ranged from 8.8 mg/dL to 10.4 mg/dL; beyond this range was defined as abnormality (28).

### 2.4 Covariates

Covariates for this study included age, gender, race/ethnicity, education attainment (EA), the ratio of family income to poverty (PIR), body mass index (BMI), time of sample collection, sun-protective behaviors and time of outdoor activity. Data were collected through household interviews and physical examinations in MEC. Age, gender, race/ethnicity, EA, PIR, sun-protective behaviors and time of outdoor activity were collected through a face-to-face interview using a structured questionnaire. Body weight and height were measured at MEC. BMI was calculated as body weight (kg) divided by body height squared (m^2^) and categorized as normal weight or below (BMI <25.0 kg/m^2^), overweight (BMI = 25.0–29.9 kg/m^2^), and obesity (BMI ≥30.0 kg/m^2^) (29). Race/ethnicity was categorized as Mexican American, other Hispanic, non-Hispanic White, non-Hispanic Black, and other race. EA was categorized as less than high school graduate, high school graduate/general educational development (GED) or equivalent, and more than high school. The NHANES collected data in the south during November–April and in the north during May–October. Sun-protective behaviors between 2009 and 2018 were collected from three questions: staying in the shade, wearing long-sleeved shirts, and using sunscreen. Responses to these questions were “always,” “most of the time,” “sometimes,” “rarely,” and “never.” Sun-protective behaviors were classified as rare (never or rarely), moderate (sometimes), and frequent (always or most of the time); they were scored as 1, 2, or 3, respectively. According to a previous study (30), we categorized total scores as rare (≤5), moderate (6–7), and frequent (≥8).

### 2.5 Statistical analysis

All analyses incorporated sample weights, stratification, and clustering of the complex sampling design to ensure nationally representative estimates. The alpha level was 0.05. All statistical analyses were performed by using the SPSS software (version 24.0, IBM, Inc., New York, United States) and the R software (version: 3.4.3; R Foundation for Statistical Computing, Vienna, Austria).

We descriptively analyzed the characteristics of the study participants. For continuous variables with normal distribution, we presented the data as means ± standard errors (SEs). For continuous variables with skewed distribution, we showed the data as medians and inter-quartile ranges. For categorical variables, we presented the data as frequencies and weighted percentages. We conducted linear trend tests for continuous covariates in linear regression models and categorical variables in logistic regression models.

Means and 95% confidence intervals for calcium and vitamin D intake and circulating levels from 2007 to 2018 were estimated. Serum levels of 25(OH)D2 and 25(OH)D3 were also analyzed separately. Linear regressions were used to test for their secular trends. In the model, the dependent variables were calcium intake, serum total calcium, vitamin D intake, and serum 25(OH)D, and the independent variable was survey year. The trend of calcium intake from 2009 onwards was also analyzed because we observed a consistent declining trend for calcium intake from that year based on our prior analysis. To examine the differences in trends between calcium intake and serum total calcium, and between vitamin D intake and serum 25(OH)D, interaction tests [calcium (i.e., calcium intake and serum total calcium) *survey year and vitamin D (i.e., vitamin D and serum 25(OH)D) *survey year] were tested using linear regression models. Insignificant interaction means that the secular trends of nutrients between intake and circulating levels across survey years were paralleled. In other words, insignificant interaction indicates that there might be a correlation between nutrient intake and its circulating levels. We used logistic regression models to analyze secular trends in the prevalence of inadequate calcium and vitamin D intake, severe vitamin D deficiency, moderate vitamin D deficiency, vitamin D insufficiency, and abnormal serum total calcium. In the model, the dependent variables included inadequate calcium and vitamin D intake, severe and moderate vitamin D deficiency, vitamin D insufficiency, and abnormal serum total calcium; the independent variable was survey year. All models were adjusted for gender, age, race/ethnicity, PIR, EA, and BMI; only models for serum 25(OH)D were further adjusted for the time of sample collection. Secular trends of serum total calcium stratified by calcium intake, serum 25(OH)D, EA, and BMI groups were tested to determine whether calcium intake, 25(OH)D, EA and BMI impacted observed trends in serum total calcium. Similarly, we examined the secular trends of serum 25(OH)D stratified by groups of sun protection behaviors, vitamin D intake, EA, BMI and time of outdoor activity. An insignificant interaction between groups of a factor (*P* trend interaction between groups >0.05) indicates that the factor had a potential impact on vitamin D and calcium intake or their serum levels. Trends in calcium and vitamin D intake were further analyzed by EA and BMI. Interaction terms tests and analysis of variance were used to test for trends and mean differences in calcium and vitamin D intakes, serum total calcium and 25(OH)D levels between groups, respectively.

## 3 Results

After excluding ineligible individuals (Figure 1), we finally identified 16,751 US adults in this study. As compared with excluded participants, the included individuals were older (median age: 43.0 vs. 28.8 years; *p* < 0.001), more likely to be males (55.2% vs. 45.3%; *p* < 0.001) and had greater proportions of obese individuals (36.7% vs. 22.9%; *p* < 0.001). From 2007 to 2018, the percentage of the other race persons rose from 5.5 to 10.4% (*P* trend <0.001; Table 1). The percentage of adults with less than high school education decreased from 6.1 to 2.7% (*P* trend <0.001). The percentage of adults who were overweight declined from 36.7 to 30.8% (*P* trend =0.005), whereas the percentage of adults who were obese increased from 33.5 to 41.7% (*P* trend <0.001). Participants with ≥2 h outdoor activity increased with survey years (*P* trend <0.001). There were no significant trends across calendar years for gender, age, PIR, time of sample collection, and frequency of sun protection behaviors.

**Table 1:** Characteristics of US adults by NHANES survey cycle, 2007 to 2018.

Characteristics	2007–2008 (*n* = 2,364)	2009–2010 (*n* = 3,050)	2011–2012 (*n* = 2,704)	2013–2014 (*n* = 3,135)	2015–2016 (*n* = 2,906)	2017–2018 (*n* = 2,592)	*P* trend
Sex (*n*, weighted %)							
Male	1,304 (54.8)	1,663 (55.3)	1,452 (54.7)	1,693 (56.4)	1,542 (54.6)	1,392 (55.3)	0.854
Female	1,060 (45.2)	1,387 (44.7)	1,252 (45.3)	1,442 (43.6)	1,364 (45.4)	1,200 (44.7)	
Age, years	43.6 (31.0, 54.7)	42.7 (29.8, 55.0)	43.1 (29.7, 56.1)	42.3 (29.0, 55.9)	43.5 (30.5, 56.2)	43.1 (29.9, 56.5)	0.176
Race-ethnicity (*n*, weighted %)							
Mexican American	414 (8.5)	619 (9.5)	313 (9.2)	466 (10.3)	557 (9.8)	355 (9.0)	0.831
Other Hispanic	275 (5.0)	304 (4.9)	277 (6.8)	294 (6.0)	372 (6.4)	228 (6.5)	0.227
Non-Hispanic White	1,074 (69.4)	1,414 (67.8)	939 (64.6)	1,254 (64.0)	886 (63.0)	876 (62.8)	0.123
Non-Hispanic Black	514 (11.7)	557 (11.1)	736 (11.9)	647 (11.7)	643 (11.9)	619 (11.4)	0.968
Other race	87 (5.5)	156 (6.6)	439 (7.5)	474 (8.0)	448 (8.8)	514 (10.4)	<0.001
Education (*n*, weighted %)							
Less than high school	270 (6.1)	337 (5.7)	200 (4.8)	217 (4.4)	296 (5.1)	167 (2.7)	<0.001
High school/ (GED)	1,012 (38.8)	1,240 (36.2)	968 (31.3)	1,220 (34.5)	1,055 (31.5)	984 (37.1)	0.362
More than high school	1,082 (55.1)	1,473 (58.1)	1,536 (63.9)	1,698 (61.1)	1,555 (63.4)	1,441 (60.3)	0.070
Ratio of family income to poverty	2.6 (1.3, 4.8)	2.7 (1.3, 4.7)	2.6 (1.1, 4.7)	2.5 (1.2, 4.7)	2.7 (1.4, 4.9)	2.8 (1.4, 4.9)	0.705
Body mass index (*n*, weighted %)							
<25.0 kg/m^2^	669 (29.8)	897 (31.6)	849 (29.5)	1,008 (30.8)	847 (29.3)	720 (27.5)	0.144
25.0–29.9 kg/m^2^	851 (36.7)	1,017 (33.4)	888 (35.4)	1,008 (33.2)	946 (32.7)	812 (30.8)	0.005
≥30.0 kg/m^2^	844 (33.5)	1,136 (35.0)	967 (35.1)	1,119 (36.0)	1,113 (38.0)	1,060 (41.7)	<0.001
Time of sample collection (*n*, weighted %)							
November 1 through April 30	1,048 (35.9)	1,431 (40.2)	1,292 (45.2)	1,539 (45.1)	1,480 (44.5)	1,260 (45.9)	0.284
May 1 through October 31	1,316 (64.1)	1,619 (59.8)	1,412 (54.8)	1,596 (54.9)	1,426 (55.5)	1,332 (54.1)	
Time of outdoor activity (*n*, weighted %)							
<0.5 h	NA	349 (12.8)	339 (16.5)	379 (14.6)	318 (11.4)	195 (7.8)	0.001
0.5–2 h	NA	813 (40.8)	781 (40.0)	882 (41.2)	798 (41.3)	700 (40.5)	<0.001
≥2 h	NA	964 (46.4)	793 (43.6)	908 (44.3)	931 (47.2)	798 (51.7)	<0.001
Frequency of sun protection (*n*, weighted %)							
Rare	NA	187 (26.0)	146 (23.7)	220 (28.7)	234 (29.0)	216 (30.0)	0.053
Moderate	NA	316 (53.8)	303 (49.7)	341 (49.5)	411 (51.3)	334 (51.1)	0.721
Frequent	NA	120 (20.2)	129 (26.6)	152 (21.8)	144 (19.7)	148 (18.9)	0.174

GED, general equivalency diploma. Variables with a normal distribution are shown as mean ± standard error; variables with a skewed distribution are shown as median (inter-quartile range); categorical variables were presented as *n*, (%). *P* trend was adjusted for NHANES survey weights to be nationally representative.

**Figure 2:**
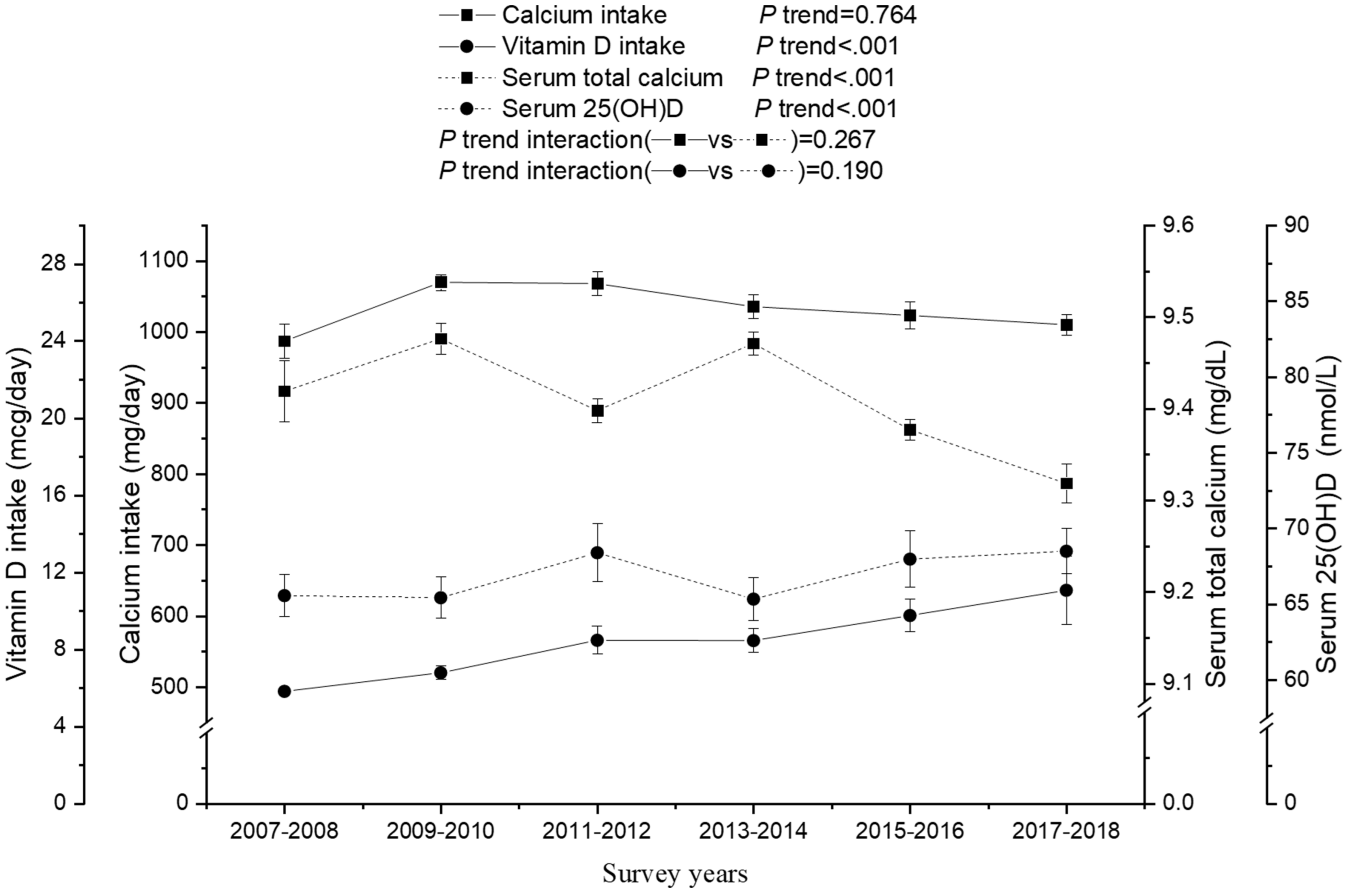
Secular trends of calcium and, vitamin D intake, serum total calcium, and 25(OH)D concentrations among US adults across the NHANES survey cycle, 2007–2018. Dots represent means and error bars stand for 95% confidence intervals. *P* trend interaction was adjusted for the age, gender, race/ethnicity, education attainment, the ratio of family income to poverty, body mass index, and serum 25(OH)D was additionally adjusted for the time of sample collection (only for serum 25(OH)D).

Overall, serum total calcium decreased significantly during the survey years (*P* trend <0.001; Figure 2). Although the overall secular trend of calcium intake between 2007 and 2018 was insignificant (*P* trend =0.764), its trend between 2009 and 2018 was significant (1070.0 to 1010.1 mg/day; *P* trend <0.001). Vitamin D intake and serum 25(OH)D increased from 2007 to 2018 (5.8 to 11.0 mcg/day and 65.6 to 68.5 nmol/L, respectively; all *P* trend <0.001). There were similar trends of calcium intake and serum total calcium (*P* trend interaction =0.267), and similar trends of vitamin D intake and serum 25(OH)D with survey years (*P* trend interaction =0.190). As shown in Supplementary Table 1, although the secular trend of the prevalence of inadequate calcium intake between 2007 and 2018 was insignificant (*P* trend =0.419), its trend between 2009 and 2018 was significant (*P* trend <0.001). The prevalence of inadequate vitamin D intake decreased significantly from 86.0 to 80.2% (*P* trend =0.002). The prevalence of severe vitamin D deficiency and vitamin D insufficiency showed insignificant trends, whereas the prevalence of moderate vitamin D deficiency decreased from 22.3 to 21.5% (*P* trend =0.043). There was little change in the prevalence of abnormal serum total calcium (*P* trend =0.430).

**Figure 3:**
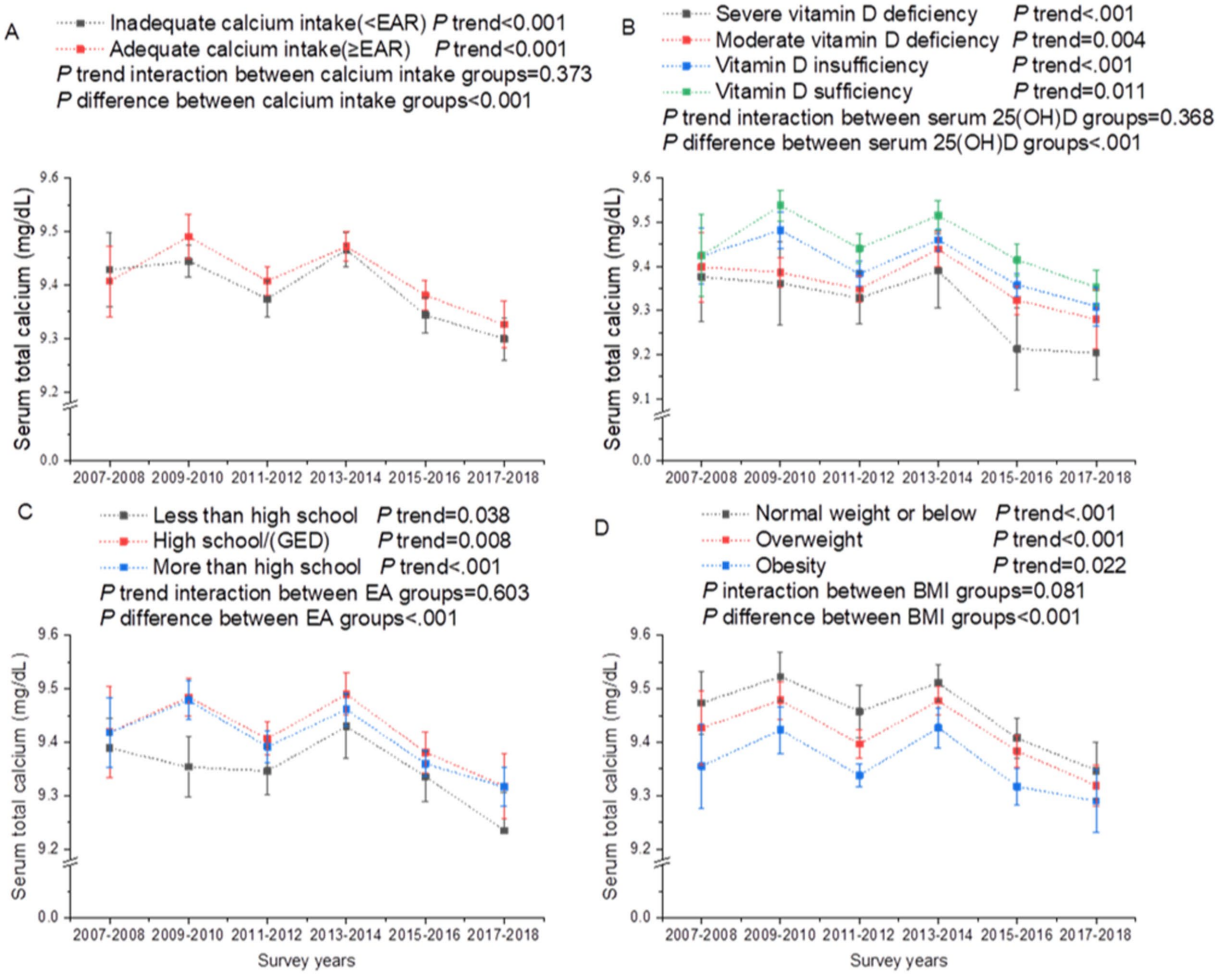
Secular trends of serum total calcium among US adults across the NHANES survey cycles, 2007–2018. **(A)** Secular trends of serum total calcium by calcium intake. **(B)** Secular trends of serum total calcium by serum 25(OH)D concentration. **(C)** Secular trends of serum total calcium by education attainment. **(D)** Secular trends of serum total calcium by body mass index. Dots represent means and error bars stand for 95% confidence intervals. *P* trend interaction and *P* difference were adjusted for age, gender, race/ethnicity, education attainment, the ratio of family income to poverty, and body mass index.

**Figure 4:**
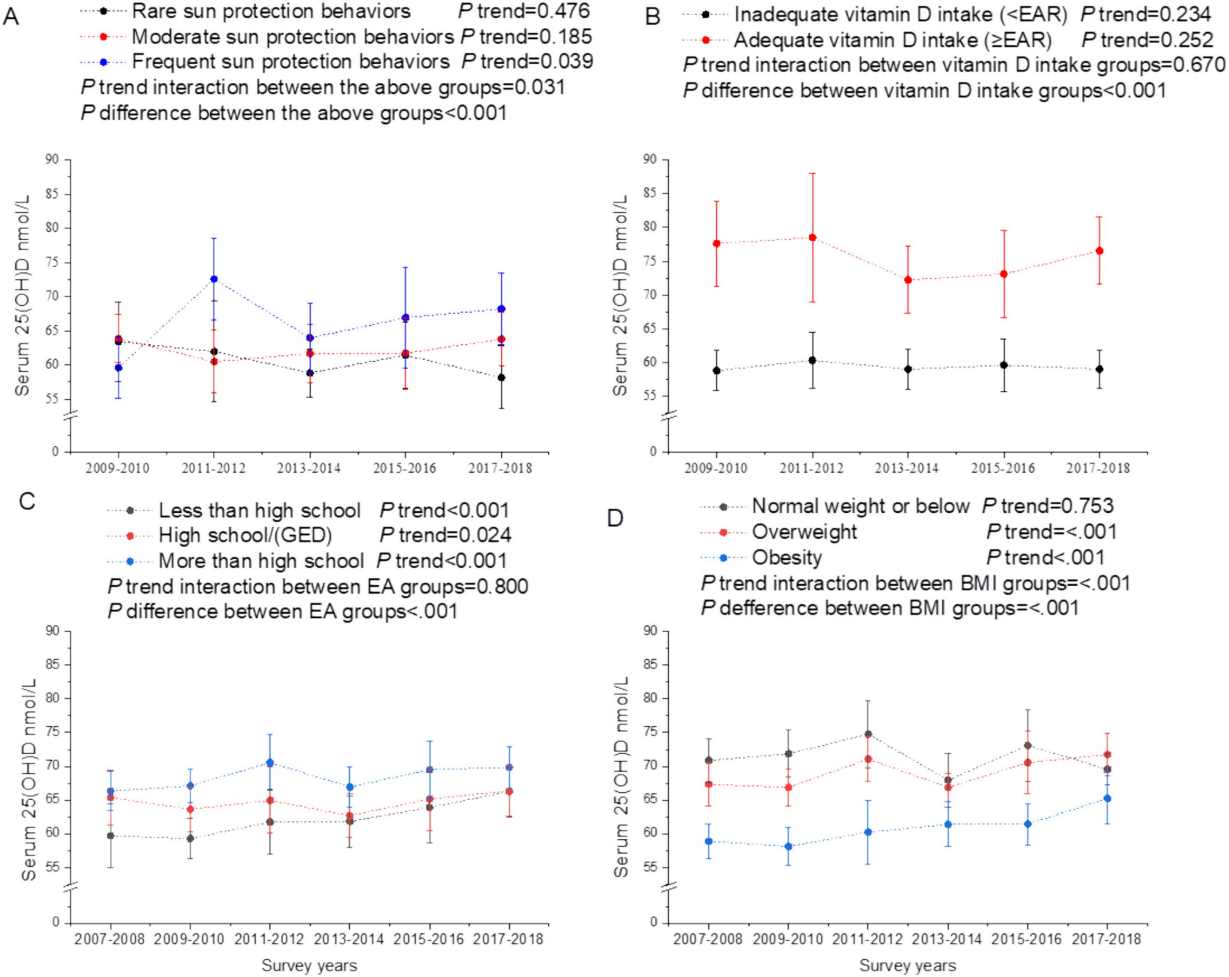
Secular trends of serum 25(OH)D concentrations among US adults across the NHANES survey cycles, 2007–2018. **(A)** Secular trends of serum 25(OH)D concentrations by sun-protection behaviors, **(B)** Secular trends of serum 25(OH)D concentrations by vitamin D intake. **(C)** Secular trends of serum 25(OH)D concentrations by education attainment. **(D)** Secular trends of serum 25(OH)D concentrations by body mass index. Dots represent means and error bars stand for 95% confidence intervals. *P* trend interaction and *P* difference were adjusted for age, gender, race/ethnicity, education attainment, the ratio of family income to poverty, body mass index, and the time of sample collection (for serum 25(OH)D only).

In subgroup analyses of trends in serum total calcium stratified by calcium intake, serum 25(OH)D, EA, and BMI, we noted that trends of serum total serum calcium in all groups were consistent with that in the total population (all *P* trend interaction >0.05; Figure 3). The declined trend of serum total calcium with calendar years paralleled with a decreased trend of calcium intake (between 2009 and 2018) and an increase of BMI (Figure 3). The increased levels of serum 25(OH)D with survey years were likely to be mainly driven by decreased trends of individuals receiving less than high school, and increased trends of vitamin D intake (Figure 4). The calcium and vitamin D intake trends with survey years among EA, BMI and race groups were comparable (all *P* trend interaction >0.05; Supplementary Figures 1–3). Participants with higher EA had higher calcium and vitamin D intake across calendar years (Supplementary Figure 1). Obese individuals tended to have lower calcium intake (Supplementary Figure 2). Non-Hispanic White had greater calcium and vitamin D intake than all other racial groups (Supplementary Figure 3).

Serum vitamin D levels were higher among participants with longer time of outdoor activity (Supplementary Figure 4). The elevated trend of serum vitamin D levels across survey years was primarily attributed to the trend of serum 25(OH)D3, but not 25(OH)D2 (Supplementary Figure 5). Stratified analysis by BMI showed identical results (Supplementary Figure 6).

## 4 Discussion

In this cross-sectional study, we investigated secular trends of calcium and vitamin D intake, and serum total calcium and 25(OH)D levels among US adults from 2007 to 2018. Although the overall secular trend of calcium intake was insignificant, calcium intake declined from 2009 onwards. There was a decreased trend in serum total calcium and increased trends in vitamin D intake and serum 25(OH)D from 2007 to 2018. The secular trends of serum 25(OH)D3, but not 25(OH)D2, had identical trends with overall serum 25(OH)D levels. Overall, the decreased secular trend of serum total calcium was not paralleled with the increasing trend of serum 25(OH)D.

Rooney et al. found an inversed U-shaped trend of calcium supplementation from 1999 to 2014, peaking in 2007–2008 (14). Another 5-year follow-up study found a decreasing trend of daily calcium intake among Minneapolitans during the transition to young adulthood from 1999 to 2014 (31). These findings support our results that there was a decreased calcium intake from 2009 onwards. Yu et al. discovered that calcium intake among the US population showed a significant upward trend from 1999 to 2018 (11). This trend was also noted in our research. The increasing trends of vitamin D intake and serum 25(OH)D from 2007 to 2018 in our study is in line with previous studies, in which there was an increase in the use of vitamin D supplements and a decreasing trend of vitamin D deficiency and insufficiency in the US population in 1999–2012 (15).

There were insignificant interactions for calcium intake vs. serum calcium levels and vitamin D intake vs. serum 25(OH)D levels in our study. These results suggested that greater intakes of calcium and vitamin D play an important role for increasing their circulating levels. This is consistent with a previous study that adequate nutrient intake improves its endogenous levels in humans (32). Sufficient circulating vitamin D and calcium levels have beneficial effects on skeletal and non-skeletal disorders (33).

Dairy products are an important source of calcium nutrition. A study showed that milk consumption, including both beverage milk and cereal milk, declined among U.S. adults between 2003 and 2018 (34). Other race in our study almost doubled from 2007 to 2018. It has been shown that dairy consumptions in Asians (a major population for other race in US) were low (35). These findings might explain the decreased trend in calcium intake from 2009 to 2018. The declining trend of serum total calcium with calendar years was mainly due to a decrease in calcium intake (2009–2018) and an increase in BMI. Calcium intake showed a significant negative association with BMI (36). Serum calcium levels tended to be lower among those with higher BMI. This is likely due to the fact obesity has a positive effect on insulin levels and the insulin increases postprandial urinary calcium excretion (37).

The increased trend of serum 25(OH)D with survey years might be mainly driven by a decrease in the percentage of individuals receiving less than high school and an increase in vitamin D intake. Studies have shown that higher vitamin D intake tended to be associated with higher serum 25(OH)D (38). People with lower levels of education were less likely to use vitamin D supplements due to their lower socio-economic status and weaker health awareness (39, 40). The increasing secular trend of vitamin D intake might be due to the recommendation of dietary guidelines to increase vitamin D intake from 2010 onwards (41–43).

A study has shown that higher BMI was associated with lower serum 25(OH)D (44), which was consistent with our findings. Possible explanations are that individuals with high BMI had reduced sun exposure due to limited activity and/or reduced outdoor physical activity (45), and that serum 25(OH)D is a fat-soluble compound that chelates in adipose tissue and is difficult to release into the blood (46). Our study indicated that serum 25(OH)D levels in overweight and obesity increased from 2007 to 2018; this was correlated with the elevated trends of vitamin D intake. Thus, despite the increase in the percentage of obese and the negative correlation between BMI and 25(OH)D, the serum levels of 25(OH)D increased.

Time of blood sample collection showed insignificant trends with survey years 2007–2018 in our research. Therefore, time of blood collection unlikely had an impact on the secular trends of serum 25(OH)D levels. Possible reasons for this insignificant result may be due to the fact that only two categories of months (November–April vs. May–October) were tested in our study and the NHANES collected blood samples in the south during November–April and in the north during May–October. Nevertheless, we found that time of outdoor activity had a positive relationship with serum 25(OH)D levels. The time of outdoor activity has seasonable variations. Thus, consistent with the previous results (47), our results still partly confirm that the seasons have an effect on circulating vitamin D levels.

In our study, inadequate vitamin D intake has dropped from 86 to 80% within approximately 10 years. In contrast, percent’s of US adults with inadequate calcium intake (approximately 40%) had maintained at a relatively stable level. There is still a large gap between inadequate vitamin D and calcium intake. Adequate vitamin D and calcium intake are important for promoting bone health (33). Thus, healthcare providers and policymakers should take public health measures to develop and implement prevention strategies for promoting calcium and vitamin D supplements, especially for vitamin D supplementation.

Our study has several advantages. First, this analysis is based on the most recent, large, and nationally representative population sample from the NHANES, which made the results more extrapolative. Second, calcium and vitamin D intake considered dietary intake and dietary supplements to more accurately represent the level of calcium and vitamin D intake in the population; a harmonized LC–MS method was used for the detection of 25(OH)D throughout the entire period. However, some limitations should be acknowledged. Due to the ecological study design, we did not track the individual changes of vitamin D and calcium intake, and their circulating levels across survey years. This limits our ability to make a causal inference between intake and circulating levels. The cross-sectional design in nature also precluded conclusions on how factors contributed to these trends. Factors such as chronic malnutrition, smoking and alcohol intake were not considered in this research. Potential residual confounding cannot be fully excluded. Due to the significant difference between included and excluded participants, our study was subject to potential selection bias. Lastly, we cannot exclude the potential recall bias and underreporting of 24-h recalls (48).

## 5 Conclusion

Inadequate calcium and vitamin D intake is still prevalent in the US. From 2007 to 2018, there was a decrease in serum total calcium, and an increase in serum 25(OH)D in US adults. These results indicated that calcium and vitamin D supplementation are still warranted to prevent adverse skeletal and non-skeletal outcomes.

## Data Availability

The datasets presented in this study can be found in online repositories. The names of the repository/repositories and accession number(s) can be found at: https://wwwn.cdc.gov/nchs/nhanes/default.aspx.
