# Supplementary material for: Secular trends of vitamin D and calcium intake and their circulating levels in US adults from 2007 to 2018
Source: Front Nutr. 2025 Mar 19;12:1538019. doi: 10.3389/fnut.2025.1538019 (PMC11963807; doi:10.3389/fnut.2025.1538019)
Supplement: Supplementary file 1 [file Table_1.docx]

Supplementary Material

# Supplementary Figures and Tables

## Supplementary Table

**Supplementary Table 1. Secular trends of the prevalence of inadequate calcium and vitamin D intake, vitamin D deficiency and insufficiency, and abnormal serum total calcium among US adults across the NHANES cycles, 2007 to 2018**

|  | Survey-weighted percent (95%confidence interval) | | | | | | |
| --- | --- | --- | --- | --- | --- | --- | --- |
|  | 2007-2008 | 2009-2010 | 2011-2012 | 2013-2014 | 2015-2016 | 2017-2018 | *P* trend |
| Inadequate calcium intake | 44.4  (39.4, 49.3) | 37.6  (36.2, 38.9) | 39.9  (37.7, 42.1) | 41.5  (39.0, 43.9) | 42.1  (38.3, 45.9) | 43.1  (40.3, 45.9) | 0.419 |
| Inadequate vitamin D intake | 86.0  (83.5, 88.5) | 79.6  (77.1, 82.2) | 80.4  (77.9, 82.9) | 80.0  (78.3, 81.7) | 78.2  (74.5, 81.9) | 80.2  (77.7, 82.7) | 0.0024 |
| Serve vitamin D deficiency | 3.3 (1.9, 4.7) | 3.6 (2.5, 4.7) | 2.6 (1.6, 3.6) | 2.9 (1.8, 4.1) | 2.8 (1.8, 3.9) | 2.9 (2.2, 3.6) | 0.119 |
| Moderate vitamin D deficiency | 22.3  (19.2, 25.4) | 23.2  (19.4, 27.0) | 23.3  (18.2, 28.4) | 24.1  (20.7, 27.5) | 22.8  (18.7, 27.0) | 21.5  (17.4, 25.6) | 0.043 |
| Vitamin D insufficiency | 41.9  (39.3, 44.5) | 41.2  (38.4, 44.1) | 38.1  (35.3, 40.8) | 41.8  (39.7, 44.0) | 39.6  (37.1, 42.1) | 40.8  (37.7, 44.0) | 0.677 |
| Vitamin D sufficiency | 32.5  (28.7, 36.4) | 32.0  (27.9, 36.0) | 36.0  (29.9, 42.2) | 31.1  (27.0, 35.1) | 34.7  (29.5, 39.9) | 34.8  (29.8, 39.8) | 0.002 |
| Abnormal serum total calcium | 3.8  (1.8,5.8) | 2.4  (1.5,3.2) | 2.6  (1.7,3.5) | 1.4  (1.0,1.9) | 3.1  (1.8,4.5) | 4.6  (2.6,6.7) | 0.430 |

*P* trend was adjusted for the age, gender, race/ethnicity, education attainment, ratio of family income to poverty, body mass index, and the time of sample collection (for serum 25(OH)D only).

## Supplementary Figures

**Supplementary Figure 1. Secular trends of calcium and vitamin D intake by education attainment (EA) groups among US adults across the NHANES survey cycles, 2007-2018**

Dots represent means and error bars stands for 95% confidence intervals.

*P* trend interaction and *P* difference between EA groups were adjusted for age, gender, race/ethnicity, ratio of family income to poverty, and body mass index.

**Supplementary Figure 2.** **Secular trends of calcium and vitamin D intake by body mass index (BMI) groups among US adults across the NHANES survey cycles, 2007-2018**

Dots represent means and error bars stand for 95% confidence intervals.

*P* trend interaction and *P* difference between BMI groups were adjusted for age, gender, race/ethnicity, education attainment, and the ratio of family income to poverty.


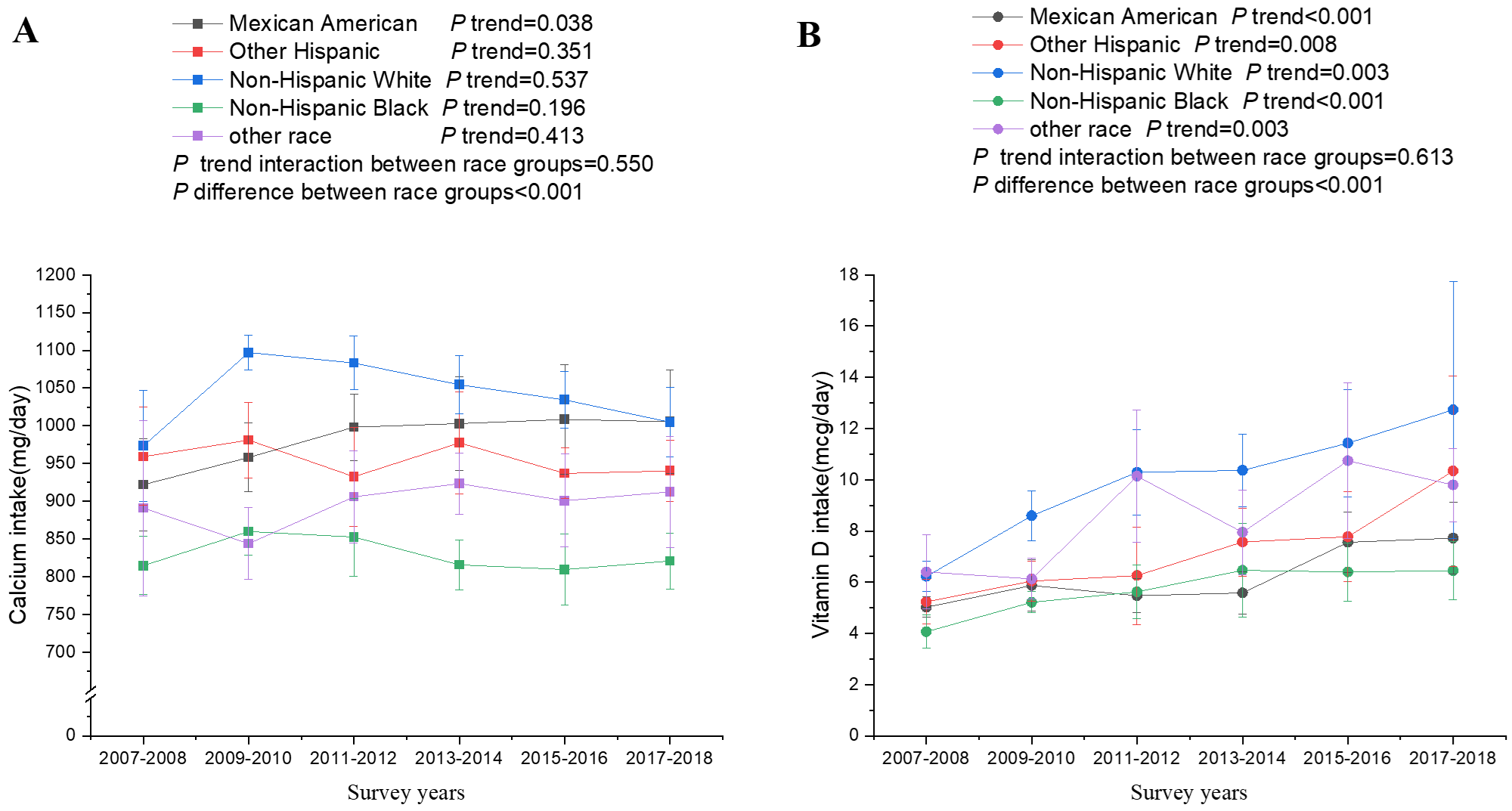


**Supplementary Figure 3.** **Secular trends of calcium and vitamin D intake by race groups among US adults across the NHANES survey cycles, 2007-2018**

Dots represent means and error bars stand for 95% confidence intervals.

*P* trend interaction and *P* difference between race groups were adjusted for age, gender, body mass index, education attainment, and the ratio of family income to poverty.


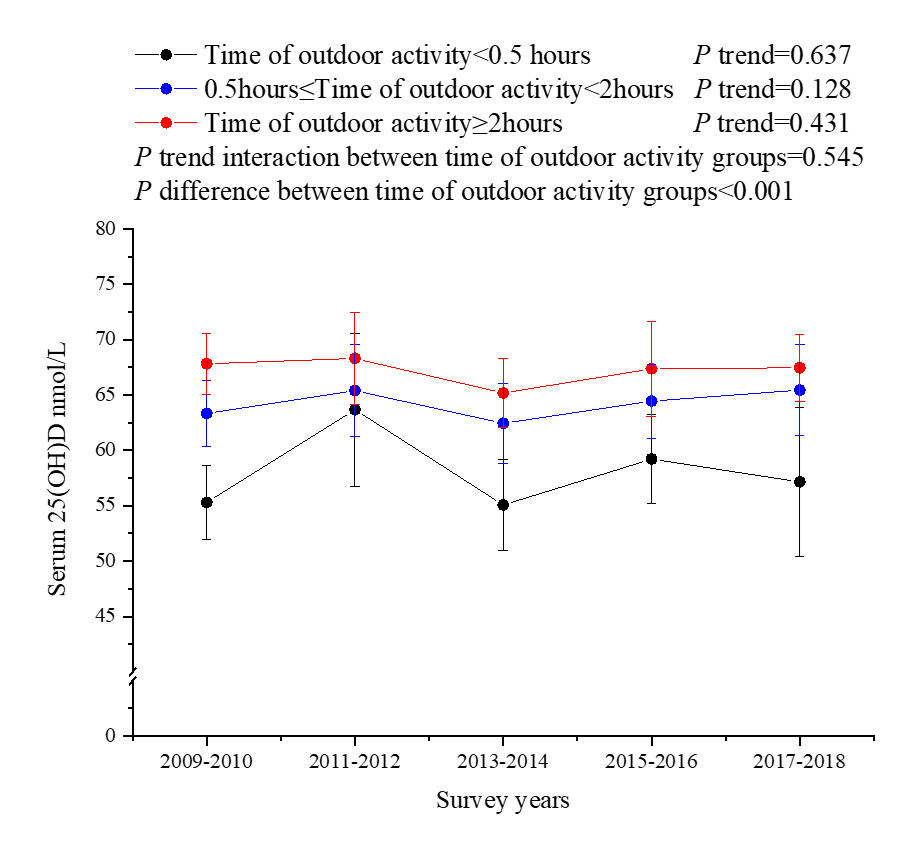


**Supplementary Figure 4.** **Secular trends of serum 25(OH)D by time of outdoor activity groups among US adults across the NHANES survey cycles, 2007-2018**

Dots represent means and error bars stand for 95% confidence intervals.

*P* trend interaction and *P* difference between outdoor activity groups were adjusted for age, gender, race/ethnicity, body mass index, education attainment, and the ratio of family income to poverty.


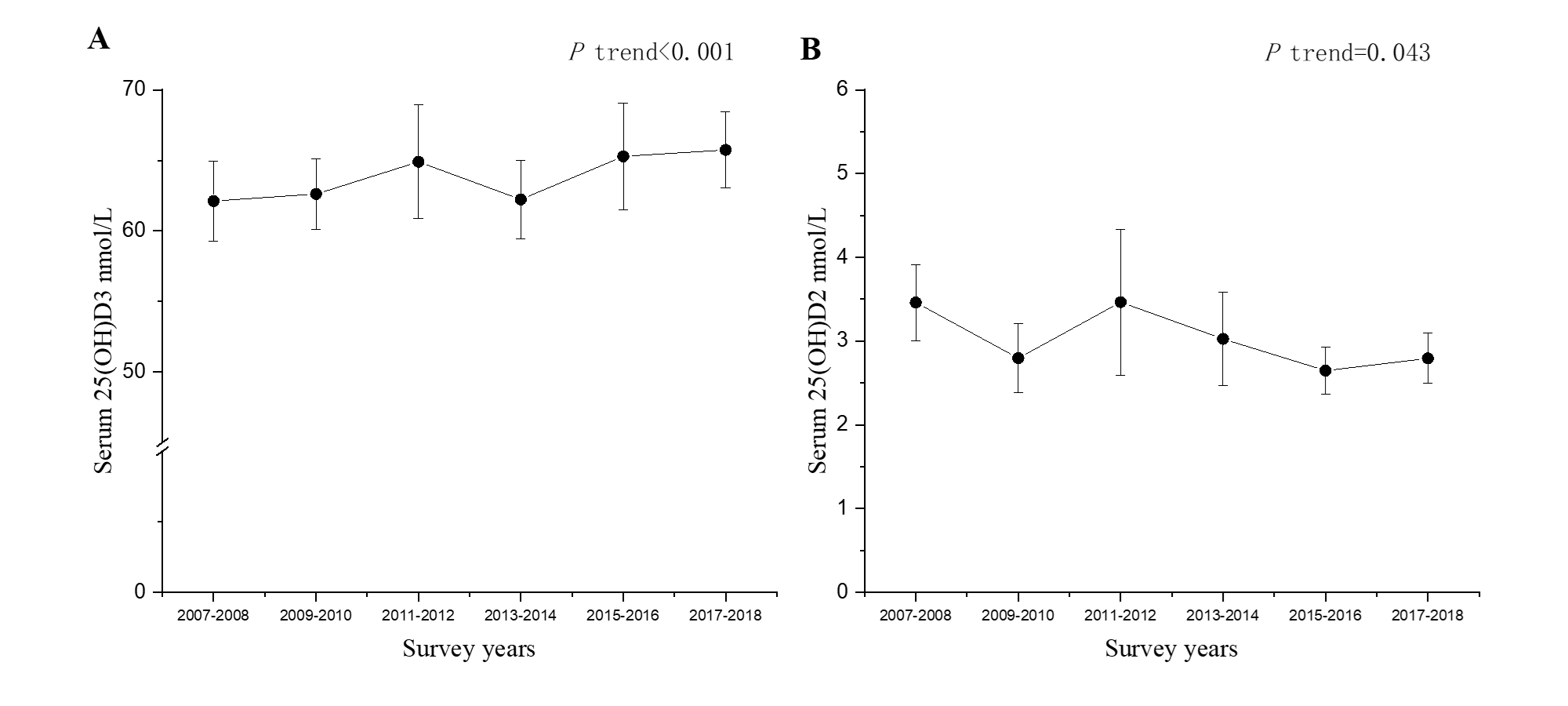


**Supplementary Figure 5.** **Secular trends of serum 25(OH)D3 and serum 25(OH)D2 among US adults across the NHANES survey cycles, 2007-2018**

Dots represent means and error bars stand for 95% confidence intervals.

*P* for trend was adjusted for age, gender, body mass index, race/ethnicity, education attainment, and the ratio of family income to poverty.


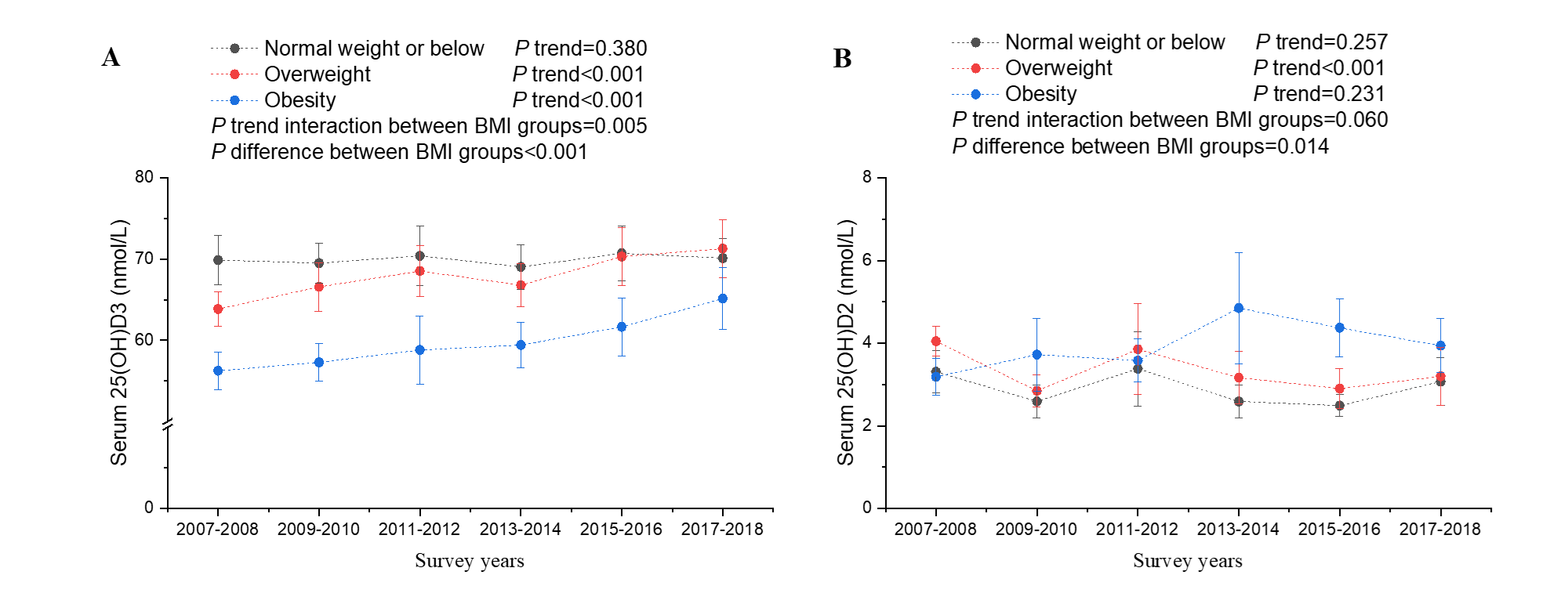


**Supplementary Figure 6.** **Secular trends of serum 25(OH)D3 and serum 25(OH)D2 by BMI groups among US adults across the NHANES survey cycles, 2007-2018**

Dots represent means and error bars stand for 95% confidence intervals.

*P* trend interaction and *P* difference between BMI groups were adjusted for age, gender, race/ethnicity, education attainment, and the ratio of family income to poverty.
